# Markers for DNA damage are induced in the rat colon by the *Alternaria* toxin altertoxin-II, but not a complex extract of cultured *Alternaria alternata*

**DOI:** 10.3389/ftox.2022.977147

**Published:** 2022-10-24

**Authors:** Georg Aichinger, Gudrun Pahlke, Hannes Puntscher, Julia Groestlinger, Stephanie Grabher, Dominik Braun, Katharina Tillmann, Roberto Plasenzotti, Giorgia Del Favero, Benedikt Warth, Harald Höger, Doris Marko

**Affiliations:** ^1^ Department of Food Chemistry and Toxicology, Faculty of Chemistry, University of Vienna, Vienna, Austria; ^2^ Laboratory of Toxicology, Department of Health Science and Technology, ETH Zurich, Switzerland; ^3^ Center for Biomedical Research, Medical University of Vienna, Vienna, Austria; ^4^ Core Facility Multimodal Imaging, Faculty of Chemistry, University of Vienna, Vienna, Austria

**Keywords:** genotoxicity, *in vivo*, emerging contaminant, food safety, mycotoxins, natural toxicant, carcinogen, epoxide

## Abstract

Mycotoxins produced by *Alternaria* spp. act genotoxic in cell-based studies, but data on their toxicity *in vivo* is scarce and urgently required for risk assessment. Thus, male Sprague-Dawley rats received single doses of a complex *Alternaria* toxin extract (CE; 50 mg/kg bw), altertoxin II (ATX-II; 0.21 mg/kg bw) or vehicle by gavage, one of the most genotoxic metabolites *in vitro* and were sacrificed after 3 or 24 h, respectively. Using SDS-PAGE/Western Blot, a significant increase of histone 2a.X phosphorylation and depletion of the native protein was observed for rats that were exposed to ATX-II for 24 h. Applying RT-PCR array technology we identified genes of interest for qRT-PCR testing, which in turn confirmed an induction of Rnf8 transcription in the colon of rats treated with ATX-II for 3 h and CE for 24 h. A decrease of Cdkn1a transcription was observed in rats exposed to ATX-II for 24 h, possibly indicating tissue repair after chemical injury. In contrast to the observed response in the colon, no markers for genotoxicity were induced in the liver of treated animals. We hereby provide the first report of ATX-II as a genotoxicant *in vivo*. Deviating results for similar concentrations of ATX-II in a natural *Alternaria* toxin mixture argue for substantial mixture effects.

## Introduction

**FIGURE 1:**
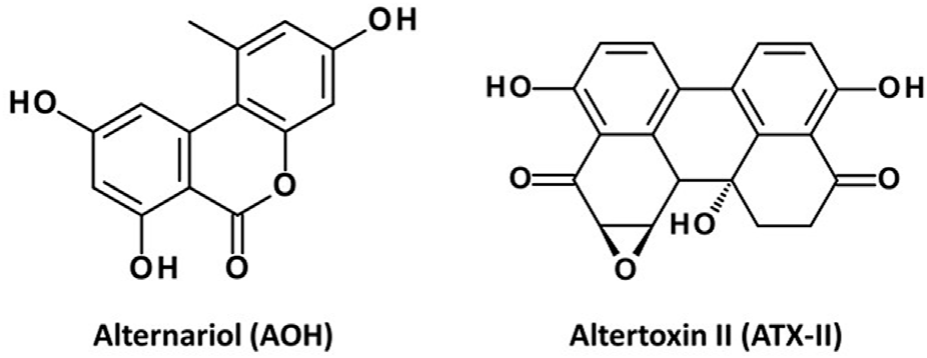
Chemical structures of AOH and ATX-II.

Fungi of the genus *Alternaria* are saprophytic organisms growing on plants, and thus play a role as contaminants of crops, feed and food. The toxicity of secondary metabolites produced by *Alternaria* spp., which can remain in affected matrices long after the producing microbes have been eliminated, has been extensively studied *in vitro*, with several compounds classified as genotoxic in human cell models (Aichinger et al., 2021). Particularly, alternariol (AOH, Figure 1) and its monomethyl ether (AME), which are also among the *Alternaria* toxins of high prevalence in commercial food samples, are well-described topoisomerase II (Top-II) poisons (Fehr et al., 2009; Jarolim et al., 2016) and are thus able to enhance the level of transient double-strand breaks in the DNA (Pfeiffer et al., 2007; Fehr et al., 2009; Tiessen et al., 2013a; Tiessen et al., 2013b; Solhaug et al., 2016). A different class of *Alternaria* metabolites, the epoxide-carrying perylene quinones with altertoxin-II (ATX-II, Figure 1) as a signature compound, by far exceed the genotoxic potential of AOH and AME (Fleck et al., 2012; Schwarz et al., 2012). These chemicals directly alkylate guanine and possibly also cytosine bases of the DNA (Soukup et al., 2020), leading to bulky adduct formation and thus extensive DNA damage *in vitro* (Fleck et al., 2012; Schwarz et al., 2012; Fleck et al., 2016). Even as ATX-II was recently for the first time described in food sample (Puntscher et al., 2020), there are doubts whether epoxy-carrying perylene quinones would be able to even reach the gastrointestinal tract due to their limited stability that is probably related to their chemical reactivity with co-occurring food constituents (Aichinger et al., 2018; Aichinger et al., 2022).

The toxicological characterization and risk assessment of *Alternaria* mycotoxins still faces scientific challenges that have so far prevented the regulation of these compounds, which are thus still regarded as “emerging” food contaminants (Fraeyman et al., 2017; Aichinger et al., 2021). First, there are gaps in occurrence data, particularly for perylene quinones that are rarely included in the methods used for food surveys (Aichinger et al., 2021). But, also for AOH and AME, human exposure levels are not fully elucidated. In 2016, the European Food Safety Authority (EFSA) estimated, that for the latter, the 95th percentile of exposure exceeds the toxicological threshold of concern but called for more occurrence data to allow a more accurate risk assessment (EFSA, 2016). Another challenge arises from the multitude of chemically diverse metabolites that are simultaneously produced by *Alternaria* spp., as well as frequently reported co-contamination with other mycotoxins, resulting in the exposure to a mixture of mycotoxins rather than isolated representatives that might lead to hardly predictable biological effects (Crudo et al., 2019). Furthermore, toxicokinetics of *Alternaria* toxins were until now mostly studied qualitatively - e.g. by assessing the type of produced human metabolites (Pfeiffer et al., 2009; Burkhardt et al., 2011; Fleck et al., 2014). The absence of quantitative toxicokinetic studies has also led to a complete lack of computational models that could be used to predict *in vivo* toxicity from *in vitro* data (Aichinger, 2021). So far, only two studies assessed absorption, distribution, metabolism and excretion of *Alternaria* toxins in animal models. Schuchardt et al. (2014) focused on AOH that was administered to mice, while Puntscher et al. (2019a) administered a complex extract (CE) obtained from an *Alternaria alternata* culture grown on rice or a corresponding dose of ATX-II to rats. In both studies, the systemic bioavailability of unconjugated mycotoxins was found to be relatively low, deeming the gastrointestinal tract and possibly the liver to be the most likely target organs for toxic effects *in vivo*. However, probably the most severe knowledge gap for risk assessment of *Alternaria* toxins is the lack of toxicity data from animal studies. So far, only one study explored genotoxic effects in mice, and this study was limited to AOH and did not take into account potential effects in the gastrointestinal tract (Schuchardt et al., 2014), a site potentially harboring carcinogenesis where the highest toxin concentrations are typically expected. Furthermore, possible combinatory effects of *Alternaria* toxins in naturally occurring mixtures were so far assessed exclusively in cellular models (Aichinger et al., 2019).

Thus, the study at hand was designed to provide a starting point for efforts to fill these gaps by investigating the genotoxic potential of ATX-II and a corresponding dose of a well characterized *Alternaria* culture extract (Puntscher et al., 2019c) *in vivo*. Sprague Dawley^®^ rats were treated per gavage, and tissue samples were analyzed with respect to topoisomerase poisoning, DNA damage and repair. Thereby, for the first time, the spotlight was put on the question whether *Alternaria* toxins in naturally occurring mixtures would cause genotoxicity in the liver or colon of mammals. In addition, we hereby provide the first *in vivo* data on the toxicity of the emerging contaminant ATX-II.

## Materials and methods

### Chemicals and reagents

Chemicals for buffers and lysis reagents were purchased from Carl Roth GmbH Co. KG (Karlsruhe, Germany) and Sigma Aldrich Chemie GmbH (Taufkirchen, Germany). Cytivia Amersham™ ECL Western Blotting detection reagents and nitrocellulose membranes were purchased from GE Healthcare (Buckinghamshire, United Kingdom). The Pierce™ BCA^®^ Protein Assay kit was obtained from Thermo Fisher Scientific (Wien, Austria).

### Antibodies

Antibodies against p53 (sc-6243), phospho-p53 (sc-101762), tubulin (sc-5286), Top-IIα (sc-365916), mouse proteins (HRP-coupled, sc-2020) and rabbit proteins (HRP-coupled, sc-2004) were purchased from Santa Cruz Biotechnology (Heidelberg, Germany), the antibodies “Phospho-Histone H2A.X (Ser139) (20E3) Rabbit mAb #9718” and “Histone H2A.X (D17A3) XP^®^ Rabbit mAb #7631” from Cell Signalling Technology (Leiden, Netherlands).

### Animal study and dosage information

The *in vivo* study was performed as previously described by Puntscher et al. (2019a) and in accordance with the Austrian animal welfare act 2012, BGBI. I Nr. 114/2012 (TVG 2012). In brief, 60 male Sprague Dawley^®^ rats were obtained from the Core Facility Laboratory Animal Breeding and Husbandry (Medical University of Vienna, Austria) and acclimated in the Center for Biochemical Research (Medical University of Vienna, Austria) for at least 14 days before starting the intervention with CE and ATX-II. Rats were randomly divided in three treatment groups, which received a single oral dose per gavage of a) the vehicle, b) CE, 50 mg/kg body weight (bw) or c) ATX-II, 0.21 mg/kg bw (corresponding to the amount of ATX-II in CE). Administered toxins were dissolved in an 1:10 (*v/v*) mixture of ethanol and sunflower seed oil, resulting in a total volume of 1 ml, with all animals receiving the same amount of ethanol (90 µL/300 g bw). The absence of *Alternaria* toxins in the solvent was confirmed as previously reported (Puntscher et al., 2019b). Animals were sacrificed 3 h or 24 h after administration by heart punctuation. Organs were quickly collected, fatty parts of tissues were removed if possible, tissue was aliquoted, kept on dry ice and finally stored at—80°C until the performance of toxicological testing. As the first three animals per group were treated with isoflurane prior sacrifice, which could cause genotoxicity (Karabiyik et al., 2001) and affect gene transcription (Bastian, 1988), the respective rat samples were excluded from analysis apart from the RT2 profiler PCR array, where pooled samples of 10 animals per group were used.

### *In vitro* complex of enzyme assay

The assay was performed based on a previously published method (Esselen et al., 2013) with slight modifications. Samples of rat colon or liver were moved to ice and supplemented with ICE lysis buffer containing 1% (*w/v*) sodium lauroyl sarcosinate in TE buffer (10 mM Tris, pH 8.0, 1 mM EDTA). Homogenization was carried out with a FastPrep-24 5G™ homogenizer (MP Biomedicals) with the following setup: Lysing Matrix D, 40 s, speed 4.0 (liver) or 6.0 (colon). Colon lysates were directly used, while for liver lysates, it was necessary to normalize the protein concentration (determined by conducting a standard bicinchoninic acid (BCA) assay according to the manufacturer’s protocol) because of the high variance in the amount of available sample material. If necessary, liver lysates were diluted with TE buffer to a protein concentration of 5 mg/ml. Afterwards, lysates were put on a cesium chloride density gradient (0.75–1.6 mg/ml) and ultracentrifugation was performed for 24 h at 100,000 g with a Thermo Scientific Sorvall™ 100 + ultracentrifuge. Afterwards, the gradient was fractionized and the fractions blotted on a nitrocellulose membrane. Subsequently, membranes were blocked in 5% (*w/v*) skim milk for 1.5 h and incubated over night with primary antibodies against anti-Top-IIα IgG (1:500 in 5% skim milk, 4°C). On the next day, the membrane was washed thrice with a Tris-buffered saline buffer containing Tween20 and incubated for 2 h at room temperature with the horse radish peroxidase (HRP) - coupled secondary antibody (1:1000 in 5% skim milk). After 3 times washing, enhanced chemiluminescence (ECL) reagents were added for 1 min, chemiluminescence was captured on the LAS-4000 system (Fujifilm Life Science, Cleve, Germany) and images were analyzed with the Fujifilm Multi-Gauge software.

### SDS-PAGE—Western blotting

Samples of rat colon or liver were supplemented with 4 ml RIPA buffer (20 mM TRIS-HCl, 150 mM NaCl, 1 mM EDTA, 1 mM EGTA, 2.5 mM sodium deoxycholate, 1% NP-40) containing 1 tablet of cOmplete™ Mini Protease Inhibitor Cocktail (Sigma-Aldrich, Schnelldorf, Germany) per 50 ml buffer as well as 1 mM sodium orthovanadate. Homogenization was carried out with a FastPrep-24 5G™ homogenizer (MP Biomedicals) with the following setup: Lysing Matrix D, 40 s, speed 4.0 (liver) or 6.0 (colon). Crude lysate (1 ml) was collected and centrifuged (10 min, 14,000 rcf, 4°C) to remove insoluble parts. The supernatant was transferred to another tube and its protein concentration was determined using BCA^®^ Protein Assay kits according to the manufacturer’s protocol. Lysates were diluted to contain 1 mg/ml protein with water and a 3x loading buffer. 20 µL of the resulting solution were loaded on a polyacrylamide gel (12%) and electrophoresis was carried out with the following gradient: 100 V for 20 min; 120 V for 30 min, 140 V for 1.5 h. Subsequently, proteins were transferred to an Amersham™ nitrocellulose membrane (pore size 0.45 µm) by wet blotting (120 V for 1.25 h). Membranes were cut and incubated with antibodies against α-tubulin, p53, phospho-p53, H2a.X or phospho-H2a.X (Ser 139) overnight at 4°C. After washing, HRP-coupled secondary antibodies were applied for 2 h at room temperature. Membranes were stained with ECL and chemiluminescence was captured on the LAS-4000 system (Fujifilm Life Science, Cleve, Germany). Images were analyzed with the Fujifilm Multi-Gauge software.

### RT^2^ profiler PCR array

To identify potential target genes involved in DNA damage and repair, the RT^2^ profiler PCR array PARN-029Z (Qiagen Hilden, Germany) was applied according to the manufacturer’s recommendations. Briefly, total RNA was extracted from liver or colon tissue samples, which have been stabilized in RNAlater (Qiagen) directly after sampling by using the RNeasy Mini Prep Kit (Qiagen). RNA was pooled from 10 animals of each study group (0.5 µg RNA/animal of control, ATX-II- and extract-treated (3 h and 24 h) group) and a total of 0.5 µg of pooled RNA was reverse transcribed into complementary DNA (cDNA) by using the RT-first strand Kit (Qiagen). The cDNA was applied for the PCR reaction setup comprising RT^2^ SYBR^®^ Green qPCR Master Mix and RNase-free water resulting in a total volume of 2.7 ml. The array plate, pre-loaded with lyophilized primers of 84 genes involved in DNA damage and repair and respective controls, was filled with 25 µl/well of PCR reaction mix. Amplification was performed according to the recommendations of the manufacturer as follows: 10 min at 95°C, 40 cycles comprising denaturation: 15 s, 95°C; annealing and extension: 1 min, 60 °C followed by a melting curve analysis. Data analysis was performed by uploading the C_t_-values in the web portal at http.//www.qiagen.com/geneglobe. Samples were assigned to controls and test groups. Normalization was conducted with the automatic selection from full panel of reference (24 h treatment) and of housekeeping (3 h treatment) genes. The geometric mean of the genes’ assay data was used as the normalization factor. The fold regulation threshold was set to 2; *p*-value: 0.05.

### Quantitative real-time PCR

For transcription analysis of the selected potential target genes *Rnf8*, *Cdc25c*, *Cdkn1a* and *Exo1* qRT-PCR was applied. Total RNA was extracted from tissue samples (30–40 mg) stabilized in RNAlater by using a FastPrep-24 5G™ homogenizer (MP Biomedicals) and the RNeasy Mini Prep Kit (Qiagen, Hilden, Germany) following the instructions of the manufacturers’ protocols. RNA purity and quantity were determined with the NanoDrop™ 2000 (Thermo Fisher Scientific, Vienna, Austria). According to the manufacturer´s protocol total RNA was reverse transcribed into complementary DNA (cDNA) using the QuantiTect^®^ reverse transcription Kit (Qiagen, Hilden, Germany). RT^2^ qPCR assays (Qiagen): *Rnf8*: PPR45635A; *Cdc25c*: PPR53710B; *Cdkn1a*: PPR06378B; *Exo1*: PPR50634A; *Actb*: PPR06570C; *Hprt*: PPR42247F and QuantiTect^®^ Sybr Green Master Mix (Qiagen) were used for gene-specific amplification with the StepOne™Plus PCR system (Applied Biosystems, Life Technologies Corporation, Carlsbad, CA, United States). The amplification protocol consisted of the initial activation of the Taq polymerase for 15 min at 95°C, 40 cycles comprising denaturation: 15 s, 94°C; annealing: 30 s, 55°C and extension: 30 s, 70 °C followed by a melting curve analysis. Relative gene transcript levels were calculated by applying the ΔΔC_t_-method as amplification efficiency was comparable. Normalization was conducted with the mean C_t_-values of the reference genes *Actb* and *Hprt*.

## Results

### Phosphorylation and depletion of histone 2a.X in colon tissue

**FIGURE 2:**
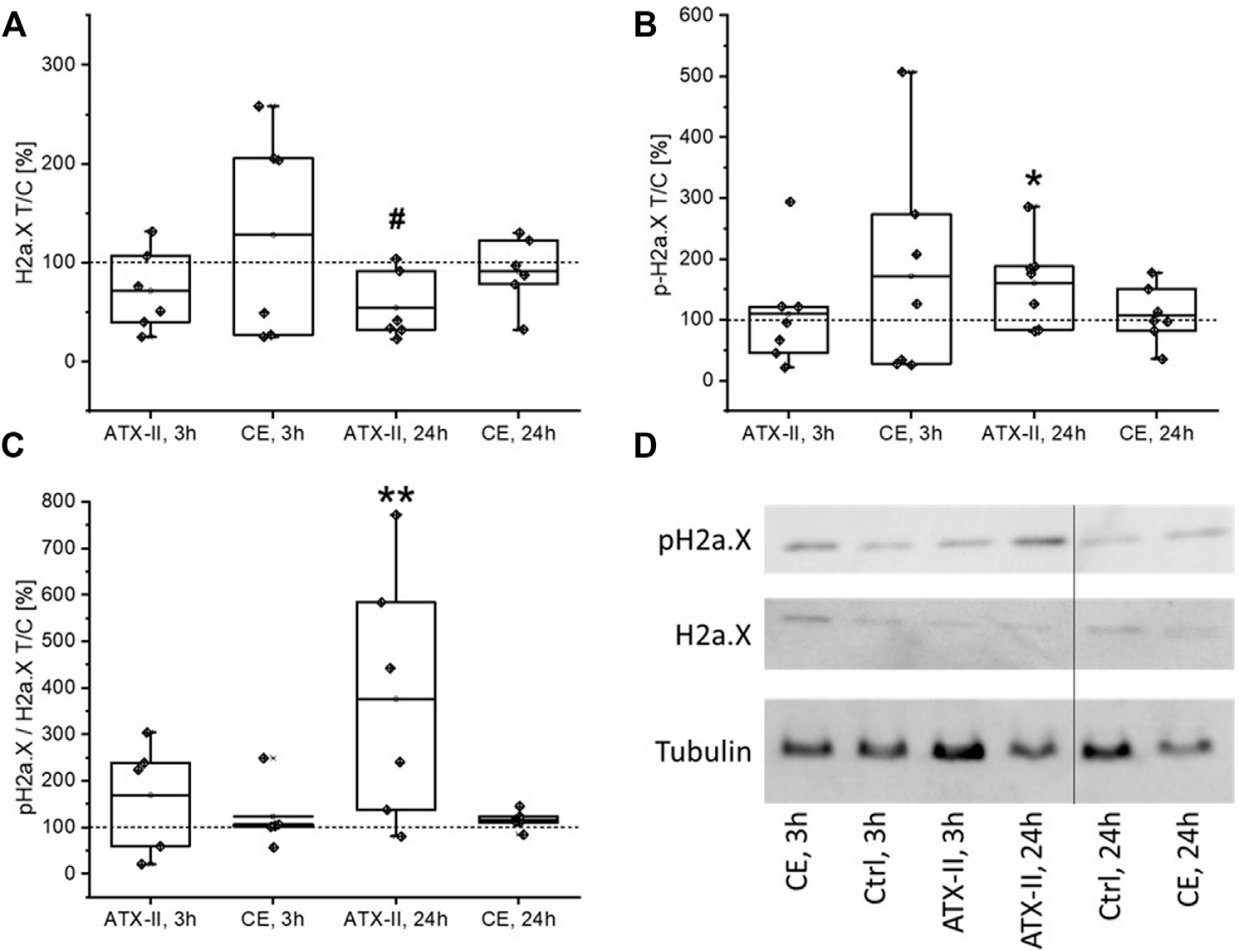
The impact of oral uptake of altertoxin-II (ATX-II) or a complex extract (CE) of *Alternaria* toxins on H2a.X phosphorylation and depletion in the colon of rats, at 3 h or 24 h after administration, as measured by SDS-PAGE/Western Blotting. **(A,B)** depict protein levels of H2a.X and its phosphorylated form, pH2a.X, respectively, as box blots and data points for the single rats. Likewise, the ratio of phosphorylated to native H2a.X levels for each rat are displayed in **(C)**. All data was normalized to the respective control (Ctrl) rat receiving the solvent that was analyzed in the same experiment, as indicated by dotted lines. Normal distribution of data was confirmed by Shapiro-Wilk testing. Significant differences to the control group were calculated using one-way ANOVA, followed by Fisher LSD post-hoc testing, and are indicated by “*” (*p* < 0.05) or “**” (*p* < 0.01). In case one-way ANOVA gave inconsistent results due to the high variability within other groups, significance testing was repeated using Student’s t-test against the control group, with positives indicated by “#” (*p* < 0.05). A representative image of membranes of one experiment is given in sub-Panel **(D)**.

The activation of H2a.X was monitored in colon tissue of rats by measuring levels of the native and the phosphorylated protein *via* SDS-PAGE/Western blotting. An influence on protein and phosphorylation levels was apparent in the colon (Figure 2). H2a.X levels were significantly decreased to 54 ± 34% of the control group in rats sacrificed 24 h after application of ATX-II (Figure 2A), while the phosphorylation of the histone (p-H2aX) was significantly enhanced to 160 ± 71% (Figure 2B), resulting in an elevated proportion of the activated protein (376 ± 241%, Figure 2C). This effect was already visible in the group sacrificed 3 h after application (p-H2a.X/H2a.X ratio of 169 ± 123%), albeit not being strong enough to be of statistical significance at any level of observation. Generally, rats treated with CE showed high variances in protein expression levels but were highly homogenous in their phosphorylation ratio (Figure 2C), suggesting that intake of the CE did not affect H2a.X activation.

### Histone 2a.X depletion and phosphorylation levels in liver tissue

**FIGURE 3:**
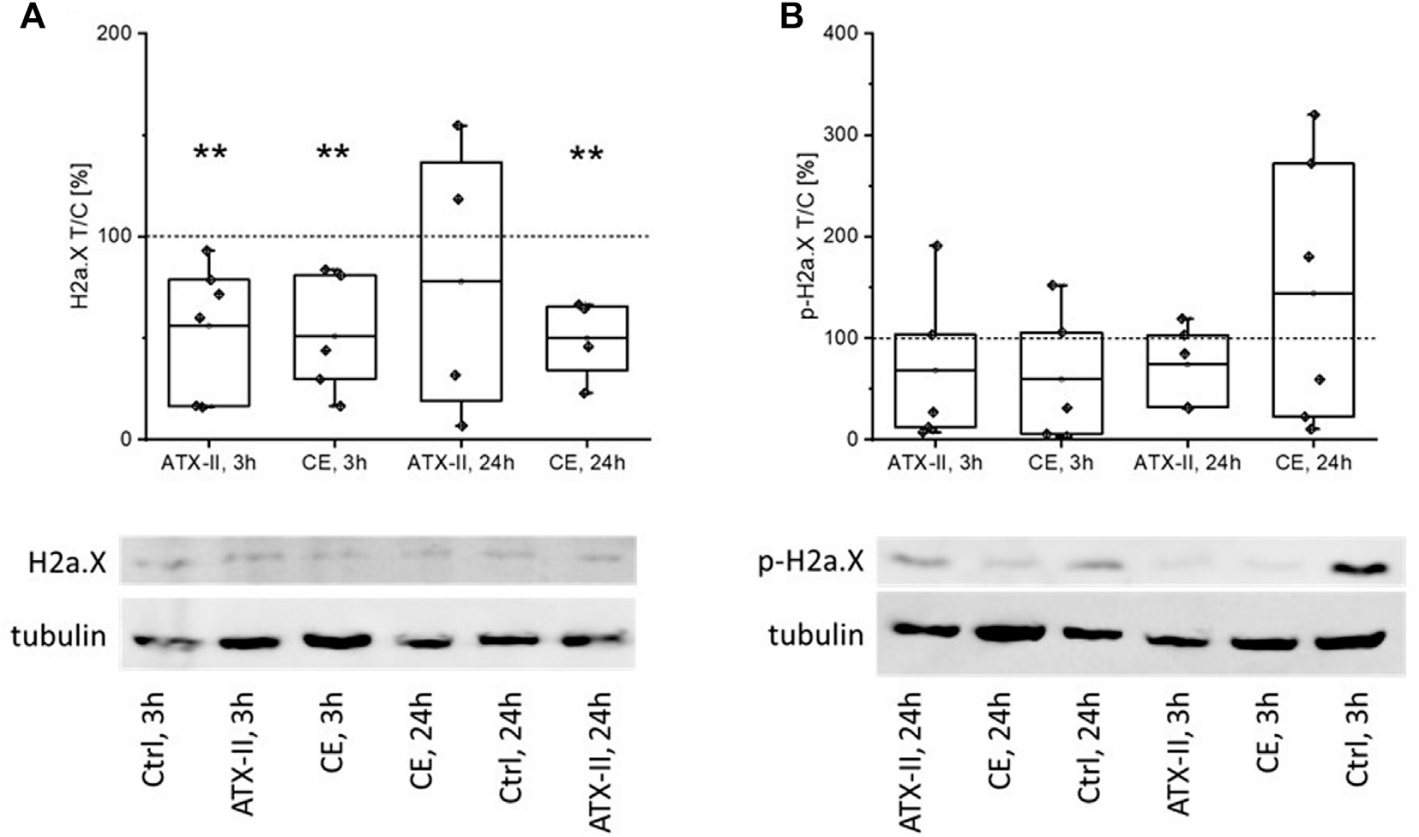
The impact of oral uptake of altertoxin-II (ATX-II) or a complex extract (CE) of *Alternaria* toxins on H2a.X phosphorylation and depletion in the liver of rats, at 3 h or 24 h after administration, as measured by SDS-PAGE/Western Blotting. **(A,B)** depict protein levels of H2a.X and its phosphorylated form, pH2a.X, respectively, as box blots and data points for the single rats. All data was normalized to the respective control (Ctrl) rat receiving the solvent that was analyzed in the same experiment, as indicated by dotted lines. Normal distribution of data was confirmed by Shapiro-Wilk testing. Significant differences to the control group were calculated using one-way ANOVA, followed by Fisher LSD post-hoc testing, and are indicated by “**” (*p* < 0.01). Directly below the graphs, representative images of Western blotting membranes are given.

Likewise, levels of native and phosphorylated H2a.X were measured in liver tissue of rats by SDS-PAGE/Western blotting. In both groups that received the CE, native H2a.X levels were significantly decreased (to 51 ± 30% after 3 h, to 50 ± 20% after 24) as compared to the control group (Figure 3A). Furthermore, a significantly lowered amount of H2a.X was measured in rats receiving ATX-II after 3 h (56 ± 33%), but not after 24 h (78 ± 70%). No significant impact of the treatment was apparent when monitoring the phosphorylation of H2a.X (Figure 3B), mainly due to the extraordinary variance within the groups. Concordantly, calculating the ratio of phosphorylated to native protein gave inconclusive results and was thus not used for data evaluation.

### Expression and phosphorylation of p53

**FIGURE 4:**
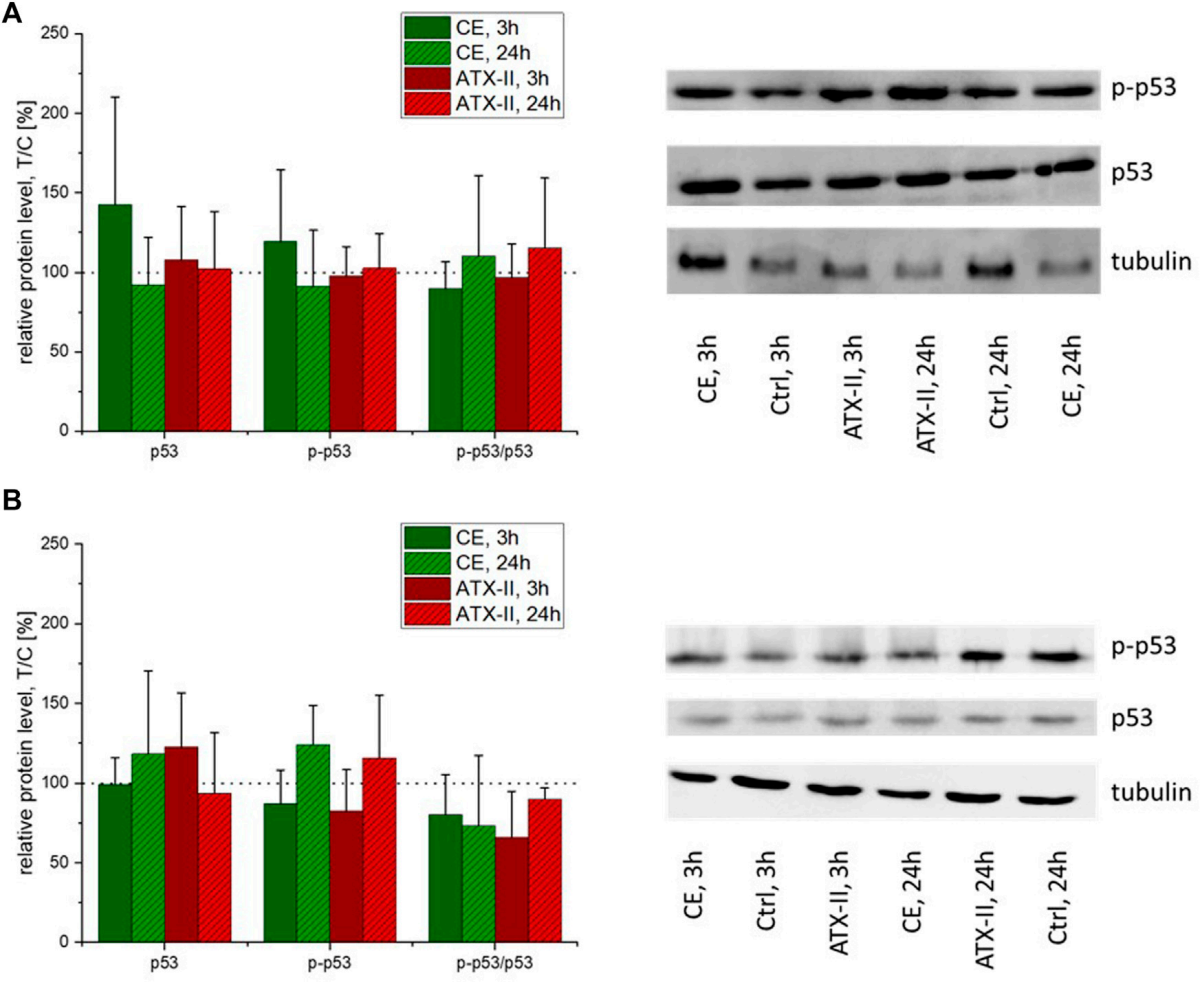
Native and phosphorylated p53 levels in **(A)** colon or **(B)** liver tissue of rats exposed to a complex extract (CE) of an *Alternaria* alternata culture or altertoxin II (ATX-II), as measured by SDS-PAGE/Western blotting. Graphs depict protein concentrations in relation to the respective vessel control group (indicated by a dotted line), as means + SD of seven animals per group. One-way ANOVA testing was carried out but revealed no significant differences (*p* < 0.05) of test groups to the respective control groups. Next to the graphs, representative images of immuno-stained Western blotting membranes are given.

Protein levels of p53 and its activated phosphate (p-p53) were measured by SDS-PAGE/Western blotting using rat colon and liver tissue. Neither treatment with the CE nor with ATX-II altered p53 or p-p53 levels in both tissues of interest (Figure 4).

### Screening for target genes

To identify target genes involved in DNA damage and repair that are potentially affected by *Alternaria* toxins, liver tissue samples were screened for changes in gene transcription applying RT^2^ profiler PCR array technology. The pooled liver RNA-samples obtained from animals that were exposed to ATX-II for 24 h led to inconclusive results as mRNA levels of an unusual high number of monitored genes were significantly reduced in comparison to the control group, suggesting impairment of validity. Thus, results from this group were not considered when selecting potentially regulated genes for further analysis. Based on the results of the remaining three test groups (ATX-II (3 h); CE (3 h, 24 h)) vs. the respective control group (Table 1), the four most promising genes were those coding for cell division cycle protein 25c (*Cdc25c,* 3.86-fold decrease of transcript levels, 3 h CE treatment), cyclin dependent kinase inhibitor 1A (p21) (*Cdkn1a*, 2.22-fold increase, 3 h CE treatment), exonuclease 1 (*Exo1*, mRNA levels increased to 37.21-fold with CE after 3 h and to 16.6-fold after 24 h ATX-II treatment) and ring finger protein 8 (*Rnf8*, 3.9-fold higher mRNA level in the CE (24 h) group than in the control group).

**TABLE 1:** Changes in gene transcription of selected genes in treated animals (10 rats per group) as determined with the RT2 profiler PCR array PARN-029Z, DNA damage and repair (Qiagen) and analyzed by uploading the Ct-values in the web portal at http.//www.qiagen.com/geneglobe. A fold-change of 2 was set as the level of significance.

Gene	Fold change transcription			Encoded protein and its function
	CE, 3 h	CE, 24 h	ATX-II, 3 h	
*Cdc25c*	−3.9	4.6	−1.7	Cell division cycle protein 25c. Regulates transition from G2 to M phase of the cell cycle, inhibition leads to arrest of cell cycle and enhanced apoptosis Cho et al. (2015)
*Cdkn1a*	2.2	1.1	1.4	Cyclin dependent kinase inhibitor 1A, also known as p21. A cofactor of p53, mediates cell cycle arrest in G1 phase as a response to DNA damage Harper et al. (1993)
*Exo1*	37	−1.1	17	Exonuclease 1. A 5′-3′ double strand DNA exonuclease critical for DNA mismatch repair Genschel et al. (2002)
*Rnf8*	−1.0	3.9	1.3	Ring finger protein 8, an E3 ubiquitin-protein ligase. Involved in non-homologous end joining, homologous recombinational and nucleotide excision DNA repair Marteijn et al. (2009); Lu et al. (2012)

### Gene transcription analysis

**FIGURE 5:**
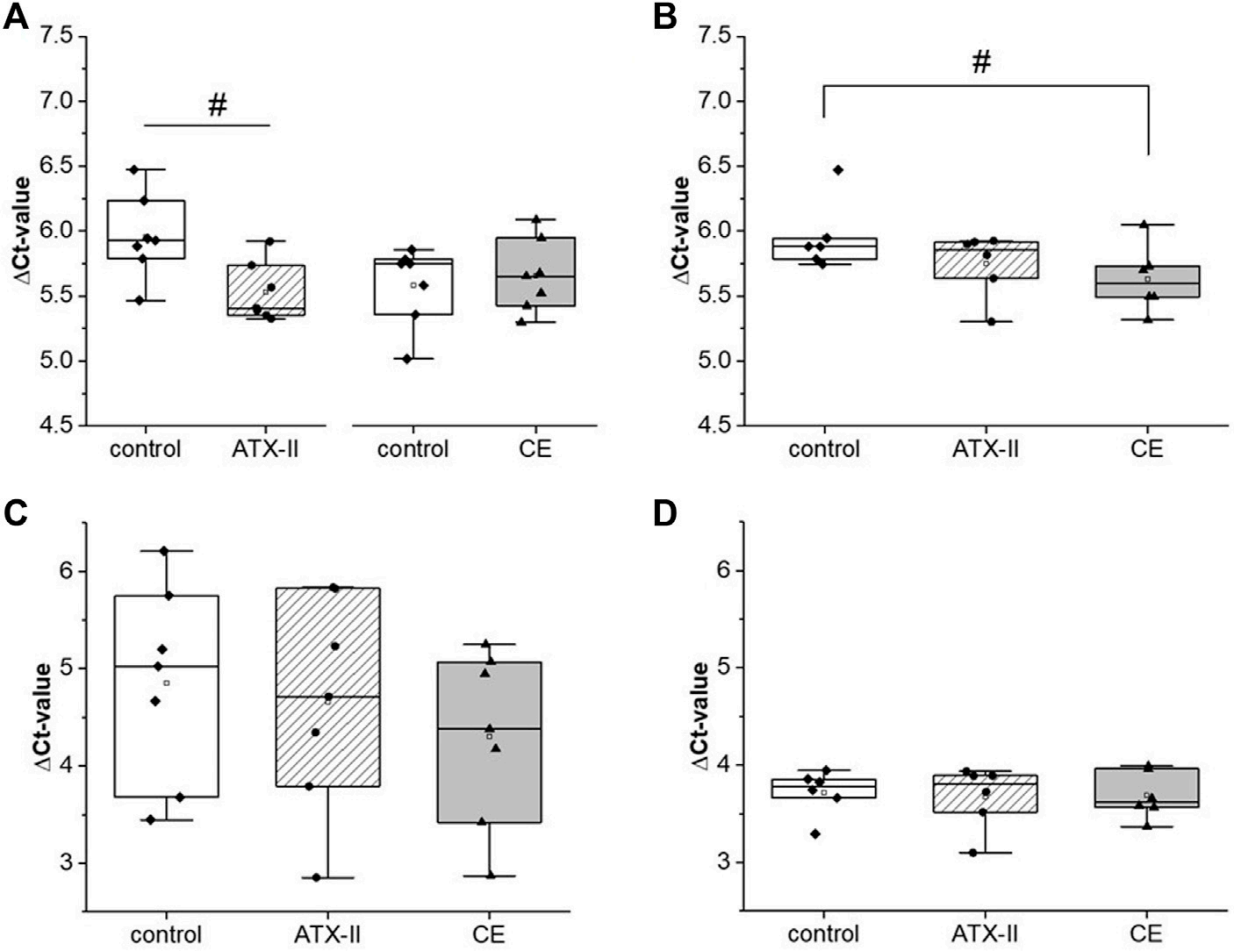
The transcription of Rnf8 in colon **(A,B)** or liver **(C,D)** tissue of rats 3 h **(A,C)** or 24 h **(B,D)** after oral uptake of either the single mycotoxin altertoxin II (ATX-II) or a complex extract (CE) of a cultured *Alternaria alternata* strain. Graphs show ΔCT values as obtained with qRT-PCR as box plots including single results (7 rats per group) of treated animals in comparison to the control group. After confirming normal distribution by the Shapiro-Wilk test, significant differences to the respective control group were calculated by one-way ANOVA, followed by Bonferroni post-hoc testing, and are indicated with “#” (*p* < 0.05).

The transcription of *Cdc25c*, *Cdkn1a*, *Exo1* and *Rnf8* was analyzed in liver and colon tissue samples of control and treatment animals by quantitative real-time PCR (qRT-PCR). Results for liver tissue barely reflected the results obtained with the RT^2^ profiler array for the pooled RNA. No impact on the selected target genes was evident with statistical significance, even though some tendencies towards increased mRNA were observed for *Cdc25c* and *Cdkn1a* 24 h after treatment. In colon tissue the picture was different. Whereas *Cdc25c* and *Exo1* were not found to be affected by exposure to *Alternaria* toxins regardless of organ or sampling time, the transcription of *Rnf8* was impacted by exposure to *Alternaria* toxins in the colon (Figure 5AB), but again not in liver tissue of rats (Figures 5C,D). 3 h after toxin application (Figure 5A), ΔC_T_ values were significantly (*p* < 0.05) decreased from 6.0 ± 0.3 (control group) to 5.5 ± 0.2 in colon tissue of rats that received ATX-II, indicating an increase in mRNA, but not among CE-receiving rats (5.7 ± 0.3 vs. 5.6 ± 0.3). After 24 h (control: ΔC_T_ = 6.0 ± 0.3), the ATX-II group was minorly affected (5.7 ± 0.2), but *Rnf8* transcription was significantly (*p* < 0.05) induced in the colon of CE-treated animals (ΔC_T_ = 5.64 ± 0.24; Figure 5B).

**FIGURE 6:**
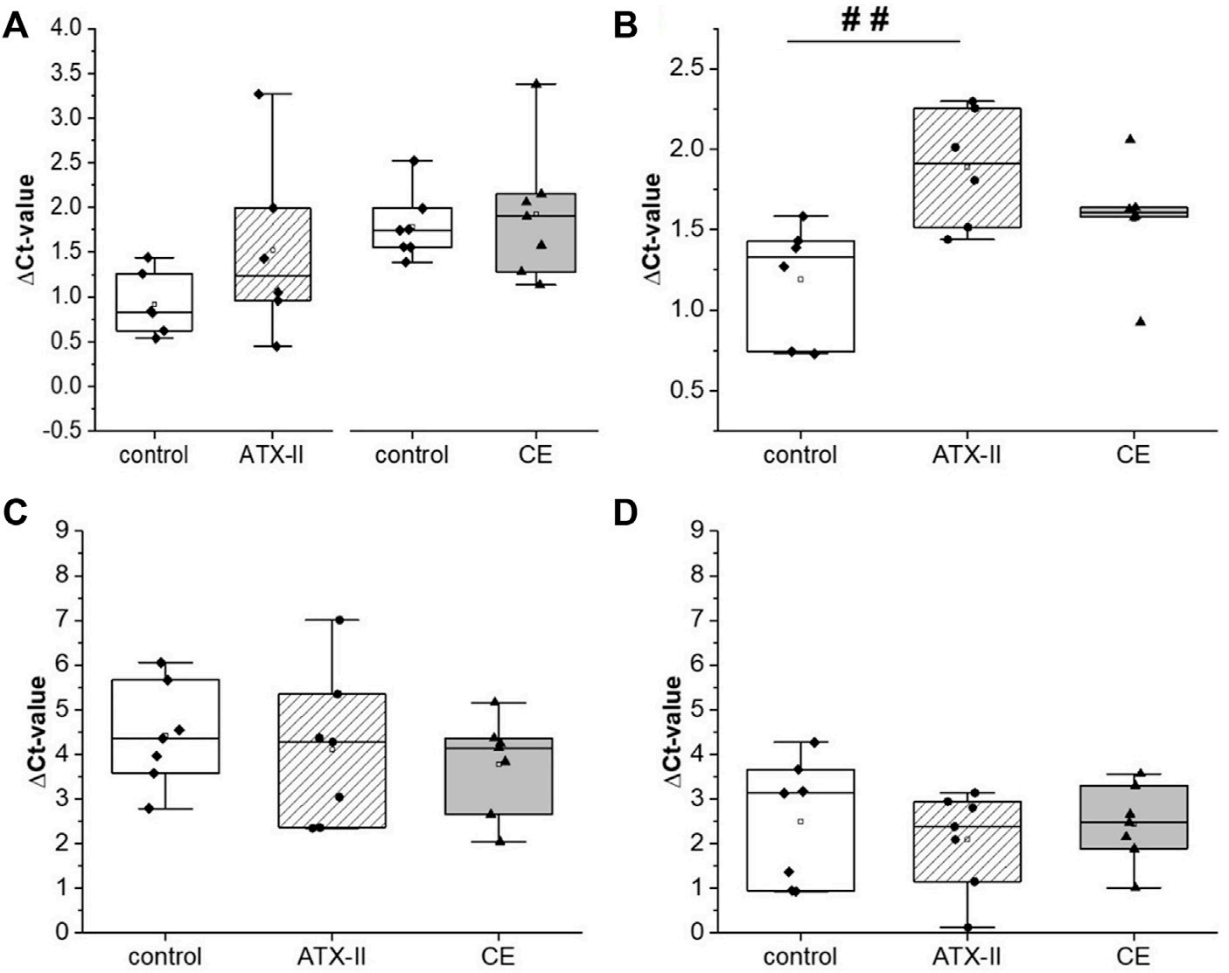
The transcription of Cdkn1a in colon **(A,B)** or liver **(C,D)** tissue of rats 3 h **(A,C)** or 24 h **(B,D)** after oral uptake of either the single mycotoxin altertoxin II (ATX-II) or a complex extract (CE) of a cultured *Alternaria alternata* strain. Graphs show ΔCT values as obtained with qRT-PCR as box plots including single results (7 rats per group) of treated animals in comparison to the control group. After confirming normal distribution by the Shapiro-Wilk test, significant differences to the respective control group were calculated by one-way ANOVA, followed by Bonferroni post-hoc testing, and are indicated with “##” (*p* < 0.01).

Furthermore, decreased mRNA levels (as indicated by increased ΔC_t_-values) of *Cdkn1a* were observed in colon tissues of rats that were exposed to ATX-II, but not to the CE. This effect was visible as a non-significant trend for animals sacrificed 3 h after gavage of the mycotoxin with a ΔC_t_-value of 1.44 ± 0.94 (Figure 6A, control: 0.92 ± 0.33), but differed significantly (*p* < 0.01) from the control (1.21 ± 0.34) after 24 h with a ΔC_t_ of 1.87 ± 0.34 (Figure 6B). No such effects were observed in liver tissue (Figures 6C,D).

### Topoisomerase poisoning

**FIGURE 7:**
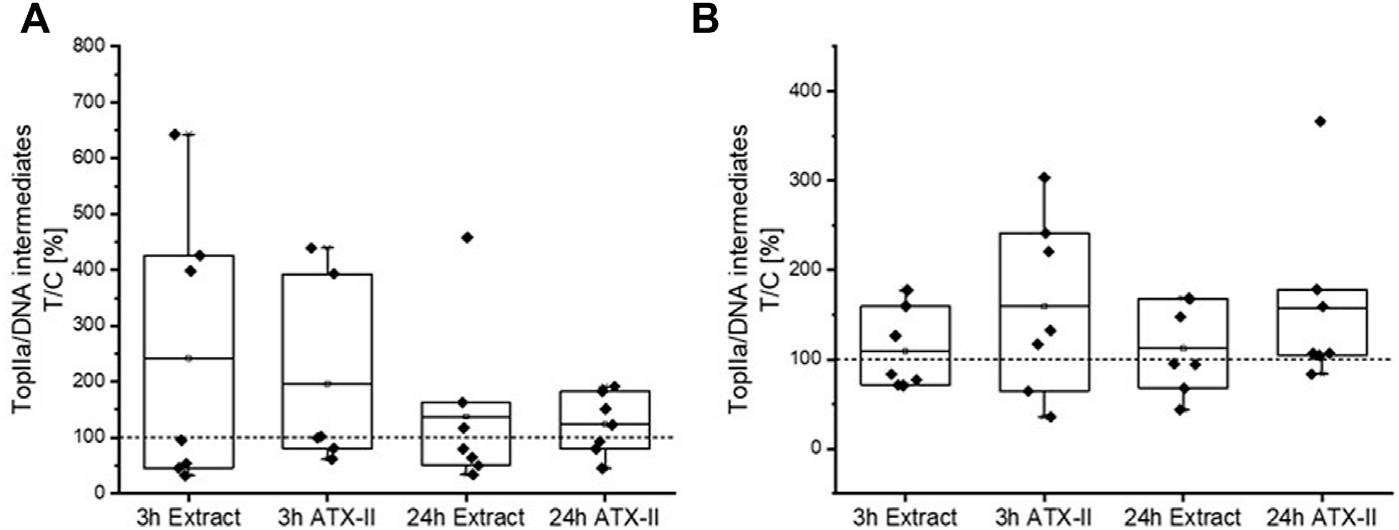
The amount of topoisomerase IIa (Top-IIα) bound to the DNA in **(A)** colon or **(B)** liver tissue of rats that were exposed to altertoxin-II (ATX-II) or to a complex extract of *Alternaria* toxins (CE). Graphs show ICE assay results of 7 rats per group, normalized to the control group (dotted lines) and as box plots including single results (black squares). Testing for significant differences to the respective control group by one-way ANOVA did not reveal positive hits.

Levels of Top-IIα covalently bound to the DNA were measured by ICE assays in both, colon and liver tissue of exposed rats. Although a strong increase of cleavable complexes was observed in some animals, particularly in the colon 3 h after toxin administration (mean values ± SD: 242 ± 244% and 196 ± 172% for CE and ATX-II, respectively), statistical evaluation revealed no significant difference to the vehicle control in either tissue (Figure 7) due to the high variation within the test groups. Furthermore, no correlation was found between Top-IIα/DNA intermediate levels in colon and liver tissue of singular rats, as well as between cleavable complex levels and H2a.X phosphorylation and/or depletion of singular rats.

## Discussion

While *Alternaria* toxins, both as isolated compounds and in naturally occurring mixtures, were described to damage the DNA in various *in vitro* test systems (Aichinger et al., 2021), the only previously conducted *in vivo* toxicodynamic study did not report any toxicity of AOH or AME in mice at comparably high doses (Schuchardt et al., 2014). Thus, the study at hand is the first to describe genotoxic effects of *Alternaria* toxins *in vivo*. In the colon of male Sprague Dawley^®^ rats, the induction of different markers for DNA damage could be observed after the application of 0.21 mg/kg bw of the epoxide-carrying perylene quinone ATX-II. The phosphorylation of H2a.X at serine 139 (yielding the phosphorylated form, γ-H2a.X) in response to DNA double strand breaks (DSBs) is a long-established biomarker for genotoxicity (Fernandez-Capetillo et al., 2004). In the colon tissue samples collected 24 h after gavage of ATX-II, a significant elevation of γ-H2a.X levels was detected by SDS-PAGE/Western blot, along with a depletion of the native protein and consequently a strong increase in the ratio of phosphorylated to non-phosphorylated H2a.X (Figure 2). With the same experimental setup, the phosphorylation of p53 was monitored as a more general marker for DNA damage. At this level, no cellular response was triggered by ATX-II (Figure 4). This discrepancy could be explained by a generally lower sensitivity of p53 as a genotoxicity marker as compared to γ-H2a.X (Bernacki et al., 2016), but also by the highly transient nature of p53 phosphorylation and nucleic accumulation in intestinal tissues (Stewart-Ornstein et al., 2021), leading to a rather small time frame in which a reaction to DNA damage could be measured. The possibility of a differential activity pattern with the time point should be taken into consideration when designing follow-up studies. Based on the results of a RT^2^ profiler PCR array screening that was conducted with pooled RNA of liver tissue samples (Table 1), four genes of potential interest were selected, and their transcript levels were measured by qRT-PCR. Of note, results regarding liver tissue gave no statistical significances, even though appropriate tendencies were observed. This might be attributed to an often occurring lack of agreement between the two methods, which has been repeatedly discussed in literature and which is the reason why a conformation of microarray results with conventional methods is regarded as necessary (Morey et al., 2006), Furthermore, the prescreening by PCR array was only conducted in a single experiment and thus no statistical analysis was possible.

Nevertheless, oral intake of *Alternaria* toxins provoked a significant effect on transcript levels of selected target genes in qRT-PCR experiments. The *Rnf8* gene was induced in the colon of rats treated with ATX-II and sacrificed after 3 h (Figure 5A), as well as in rats that received the CE and were sacrificed after 24 h (Figure 5A). The encoded ring finger protein is a major contributor to the transduction of DSB signaling and thus mediates the corresponding induction of DNA repair (Zhou et al., 2019). Also, it is directly linked to H2a.X by being responsible for its ubiquitinylation (and thus degradation) at DSB sites (Mailand et al., 2007; Marteijn et al., 2009). Thus, the measured induction of *Rnf8* transcription corresponds very well with the observed depletion of H2a.X after 24 h in the colon of ATX-II–treated rats.

Intriguingly, the transcription of *Cdkn1a* was suppressed in the ATX-II group after 24 h (Figure 6B). This gene codes for p21, an important gene product whose expression is activated by p53 and which is involved in mediating cell cycle arrest as response to DNA damage (Harper et al., 1993). While at first glance, one would rather expect its induction as a result to the DNA damage that manifested in H2a.X phosphorylation, this down-regulation might mirror an advanced stage of the response to a previous induction of transcription (and hence high present levels) of the protein as a counteracting strategy to escape cell cycle arrest after achieved DNA repair. Additionally, one might speculate that a downregulation of *Cdkn1a* could result from stem cells exiting quiescence as a reaction to repair injured tissue, a process that is heavily regulated by decreased p21 protein levels (Cheng et al., 2000; Cheung and Rando, 2013). The latter mechanism would correlate with the observation of visible enlarged Payer’s patches in treated animals, as reported in our publication on the toxicokinetic part of the study at hand (Puntscher et al., 2019a), as these indicate an inflammation of the gastrointestinal epithelium, possibly resulting from chemical-induced injury (Jung et al., 2010). Regardless of the mode of action of p21 downregulation, the inclusion of additional time points (e.g. 6, 18 h) should be encouraged for future studies to catch potential time-related effects on the transcriptional level and further clarify this phenomenon.

The non-modulation of genotoxicity markers in liver tissue (Figures 3, 4, 5C,D, 6C,D) largely confirms previously made hypotheses of the colon as primary target of genotoxicity for *Alternaria* toxins (Aichinger et al., 2021) and particularly for epoxydic perylene quinones. After all, those compounds have been demonstrated to be highly reactive with potential co-occurring food constituents (Aichinger et al., 2018), endogenous antioxidants (Jarolim et al., 2017), and cellular macromolecules (Del Favero et al., 2020; Soukup et al., 2020). Thus, it is reasonable to question whether epoxy-bearing perylene quinones might reach the liver in their native form. In line, we did not detect ATX-II or STTX-III in plasma, urine or faeces of treated rats in our pharmacokinetic survey (Puntscher et al., 2019a). Furthermore, perylene quinones were described to activate the aryl hydrocarbon receptor and might thus facilitate their own detoxification *via* phase I metabolism in liver cells (Aichinger et al., 2019; Hohenbichler et al., 2020). Of note, the applied extract only contained low concentrations of other genotoxic *Alternaria* toxins like AOH and AME which are systemically available to a low extent (Schuchardt et al., 2014; Puntscher et al., 2019a), and could thus hypothetically cause DNA damage in tissues beyond the gastrointestinal tract.

In line with these low and potentially non-effective concentrations, no significant influence of CE ingestion on topoisomerase poisoning, the proposed mode of action for the genotoxicity of AOH and AME (Fehr et al., 2009), was apparent in liver and colon as assessed by ICE assays (Figure 7). Of note, a few animals of both treatment groups had very high levels of Top-IIα/DNA complexes in the colon after 3 h. With adduct formation as the most probable mechanism of genotoxicity for ATX-II, this might point to an enhanced recruitment of TOP enzymes to the DNA to facilitate repair mechanisms (Morotomi-Yano et al., 2018) at an early stage of contact of tissue cells with strand-breaking *Alternaria* toxins.

Intriguingly, genotoxic effects of the single mycotoxin ATX-II were generally more pronounced than effects of the CE, albeit the applied dose of the toxin was matched to its concentration in the mixture. This might indicate an antagonistic influence of co-occurring *Alternaria* metabolites, which could be mediated by a multitude of distinct mechanisms. Besides direct chemical-chemical interactions, and the yet not fully elucidated bidirectional interplay with the gut microbiome (Crudo et al., 2021), a conceivable possibility would be a potentiated activation of the endogenous anti-oxidative defense system by exposure to multiple toxins. In addition to ATX-II, several other metabolites that are present in the CE, including ATX-I (Jarolim et al., 2017) and the dibenzo-α-pyrones AOH and AME (Tiessen et al., 2013a), were previously reported to interact with the nuclear factor erythroid 2–related factor 2 (Nrf2). An activation of the Nrf2 pathway ultimately leads to an increased expression of enzymes that are critical for phase II xenobiotic metabolism (Itoh et al., 1997), but also to an enhanced biosynthesis and release of cellular antioxidants like glutathione and other cysteine derivatives (Steele et al., 2013), compounds that were recently demonstrated to detoxify ATX-II *in vitro* (Jarolim et al., 2017; Aichinger et al., 2022). Thus, if ATX-II indeed is one of the driving forces behind the genotoxicity of *Alternaria* toxin mixtures, the additional interplay with Nrf2 by co-occurring metabolites could be a viable explanation for the reduced genotoxicity of the CE *in vivo*. Again, this demonstrates the general importance of considering mixture effects in risk assessment.

As recently reviewed, *Alternaria* toxins are still regarded as “emerging mycotoxins”, i.e. there is yet insufficient data on toxicity and/or human exposure to impose regulations (Aichinger et al., 2021). One of the key pieces of so far missing information is the absence of studies demonstrating toxic effects in living organisms. The study at hand is the first to describe the genotoxicity of one of these emerging food contaminants, the perylene quinone ATX-II, in the colon of male Sprague Dawley^®^ rats. This will hopefully lead to intensified research to assess further single compounds or mixtures *in vivo* and might even develop to be a gamechanger for the question of whether *Alternaria* toxins should be regulated and monitored.

## Data Availability

The raw data supporting the conclusions of this article will be made available by the authors, without undue reservation.
